# Comparative Phylogeography of Direct-Developing Frogs (Anura: Craugastoridae: *Pristimantis*) in the Southern Andes of Colombia

**DOI:** 10.1371/journal.pone.0046077

**Published:** 2012-09-25

**Authors:** Juan C. García-R, Andrew J. Crawford, Ángela María Mendoza, Oscar Ospina, Heiber Cardenas, Fernando Castro

**Affiliations:** 1 Department of Biology, Universidad del Valle, Cali, Colombia; 2 Department of Biological Sciences, Universidad de los Andes, Bogotá, Colombia; 3 Smithsonian Tropical Research Institute, Panamá, República de Panamá; University of Massachusetts, United States of America

## Abstract

The Andes of South America hosts perhaps the highest amphibian species diversity in the world, and a sizable component of that diversity is comprised of direct-developing frogs of the genus *Pristimantis* (Anura: Craugastoridae). In order to better understand the initial stages of species formation in these frogs, this study quantified local-scale spatial genetic structuring in three species of *Pristimantis*. DNA sequences of two mitochondrial gene fragments (16S and COI) were obtained from *P. brevifrons*, *P. palmeri* and *P. jubatus* at different locations in the Cordillera Occidental. We found high levels of genetic diversity in the three species, with highly structured populations (as measured by *F*_ST_) in *P. brevifrons* and *P. palmeri* while *P. jubatus* showed panmixia. Large effective population sizes, inferred from the high levels of genetic diversity, were found in the three species and two highly divergent lineages were detected within *P. jubatus* and *P. palmeri*. Estimated divergence times among populations within *P. brevifrons* and *P. palmeri* coincide with the Pleistocene, perhaps due to similar responses to climatic cycling or recent geological history. Such insights have important implications for linking alpha and beta diversity, suggesting regional scale patterns may be associated with local scale processes in promoting differentiation among populations in the Andes.

## Introduction

The Andes of South America contain the highest levels of total species richness in the world [1], [2]. The high biodiversity of tropical montane regions may be due to their old age [3], higher rates of adaptive divergence across elevation gradients [4], [5], greater opportunities for vicariant speciation [6], [7], [8] or a mixture of factors [9], [10]. Montane diversity is often spatially partitioned, i.e., beta diversity (species composition between sites) is quite high compared to the lowlands, while alpha diversity (species within a community) is relatively low [11]. The observation that sister species, at least in vertebrates, tend to be in similar habitats [8] suggests that simple vicariance models [12] could potentially account for much montane diversity compared with models invoking ecological gradients [5]. Indeed, mountain ridges and valleys have long been considered as agents of allopatric speciation by acting as effective barriers to dispersal [13].

A given geographic barrier may not have the same effect on all species, however. Organismal biology and natural history interact with landscape features to determine to what extent a potential barrier will affect dispersal [6], [14]. Over evolutionary time the context-specific dispersal capacity of an organism will determine spatial genetic patterns [15], [16]. Even congeneric species may have contrasting ecological requirements that result in distinct phylogeographic histories [17].

Using molecular data it is possible to quantify the magnitude of genetic variation within populations and patterns of gene flow among conspecific populations. Moreover, if shared past events led to the formation of current genetic patterns, then comparing phylogeographic patterns of sympatric species may enhance our understanding of the relative roles performed by the forces of climatic, geological and ecological conditions in structuring within-species genetic variation [18], [19], [20]. Molecular studies of different amphibians in the montane Neotropics have revealed high levels of genetic diversity across small geographic areas [21], [22], [23]. However, in South America and especially in Colombia, there are only a few molecular studies on genetic diversity of widely distributed montane amphibian taxa endemic to particular regions [9]. The mountains of southwestern Colombia are considered hotspots of diversity for their unique assemblages of anurans and other taxa [24]. The number of species of anurans in the Cordillera Occidental of Colombia is steadily increasing [24], with over 150 species currently found at elevations above 1,000 meters [25].

The genus *Pristimantis* (Anura: Terrarana: Craugastoridae) is the dominant component of the total amphibian fauna of Colombia, with 200 species, i.e., 27% of the country’s 750 species [26]. Nevertheless, little is known about their ecology, phylogenetic relationships [27], population genetic structure, or levels of genetic polymorphism. This study focused on three species of *Pristimantis* with distributions in the Cordillera Occidental of Colombia. *Pristimantis jubatus* [28] is found at 2,450–2,750 meters above sea level (masl) in Munchique National Park, with a total range less than 5,000 km^2^ in the department of Cauca. This species is considered to be locally abundant, inhabits forests with high (>90%) average humidity and dense vegetation cover (>80%) and appears to be intolerant of habitat modifications [28], [29]. In contrast, *Pristimantis palmeri* [30] and *P. brevifrons* [31] are sympatric in the Cordillera Occidental, ranging from the departments of Cauca to Risaralda, with elevational ranges of 900 to 2,400 masl and 1,140–3,200 masl, respectively. They are common species with apparently stable populations, most frequently found in disturbed situations, and are considered as generalist species and easily adaptable to modified landscapes.

These species were chosen because the accessibility of the area, availability of samples, their co-distribution in the Cordillera Occidental, and their contrasting ecological requirements. Thus, they should provide a useful opportunity to investigate the local historical demographic processes behind the larger scale biogeographic patterns in an ecoregion with a dynamic geologic history [32]. Information on intraspecific genetic variation is also important to inform conservation plans at the level of communities and biogeographic subregions [33], because data on population sizes and migration rates may be useful for predicting the effects of disease, climate change and isolation by habitat fragmentation on amphibian populations [34], [35], [36].

Determining the processes promoting diversification and the geographic and temporal scales at which they operate is key to understanding how this group of direct-developing frogs has attained such remarkable levels of beta diversity across the Andes mountains [27]. This study investigates the population histories on different spatial scales of three species of *Pristimantis* and focuses on the following questions: 1. Is genetic diversity randomly distributed in each species with respect to geography? 2. Do species with broad versus narrow distributions show similar patterns in the distribution of genetic variation? 3. Do sympatric species have common phylogeographic patterns because they share similar environmental histories, or do species with contrasting life histories show distinct phylogeographic patterns?

## Results

### MtDNA Diversity

The 16S alignment contained 536 base pairs (bp) for *P. brevifrons* (N = 19 individuals), and 537 bp for *P. palmeri* (N = 32) and *P. jubatus* (N = 30). This locus showed the number of segregating sites, S = 18 for *P. brevifrons*, S = 12 for *P. palmeri*, and S = 26 for *P. jubatus*. The COI alignment contained 658 bp for *P. brevifrons* (N = 16), and 618 bp for *P. palmeri* (N = 35) and *P. jubatus* (N = 31). *Pristimantis brevifrons* showed S = 30 polymorphic sites, while *P. palmeri* and *P. jubatus* contained S = 110 and S = 50 sites, respectively. The translation of amino acids using the genetic code for vertebrates showed that COI variants were synonymous (silent).

**Figure 1:**
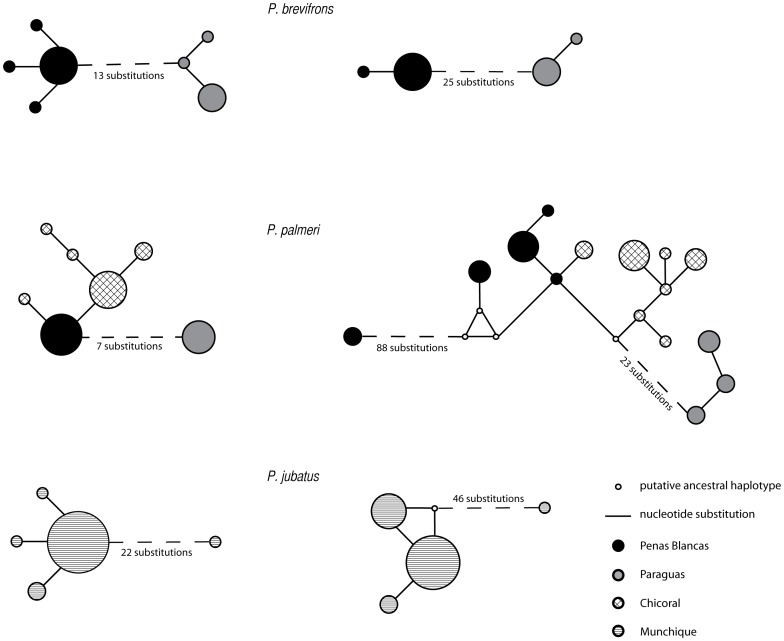
Networks of mitochondrial DNA haplotypes for 16S (left) and COI (right) from *P. brevifrons, P. palmeri* and *P. jubatus*. Branches represent inferred mutational steps, where more than three mutations are denoted by the number of substitutions. The size of the circle is proportional to the number of individuals found for each haplotype. The two highly divergent specimens from Peñas Blancas in *P. palmeri* and one from Munchique National Park in *P. jubatus* were included in the networks.

**Figure 2:**
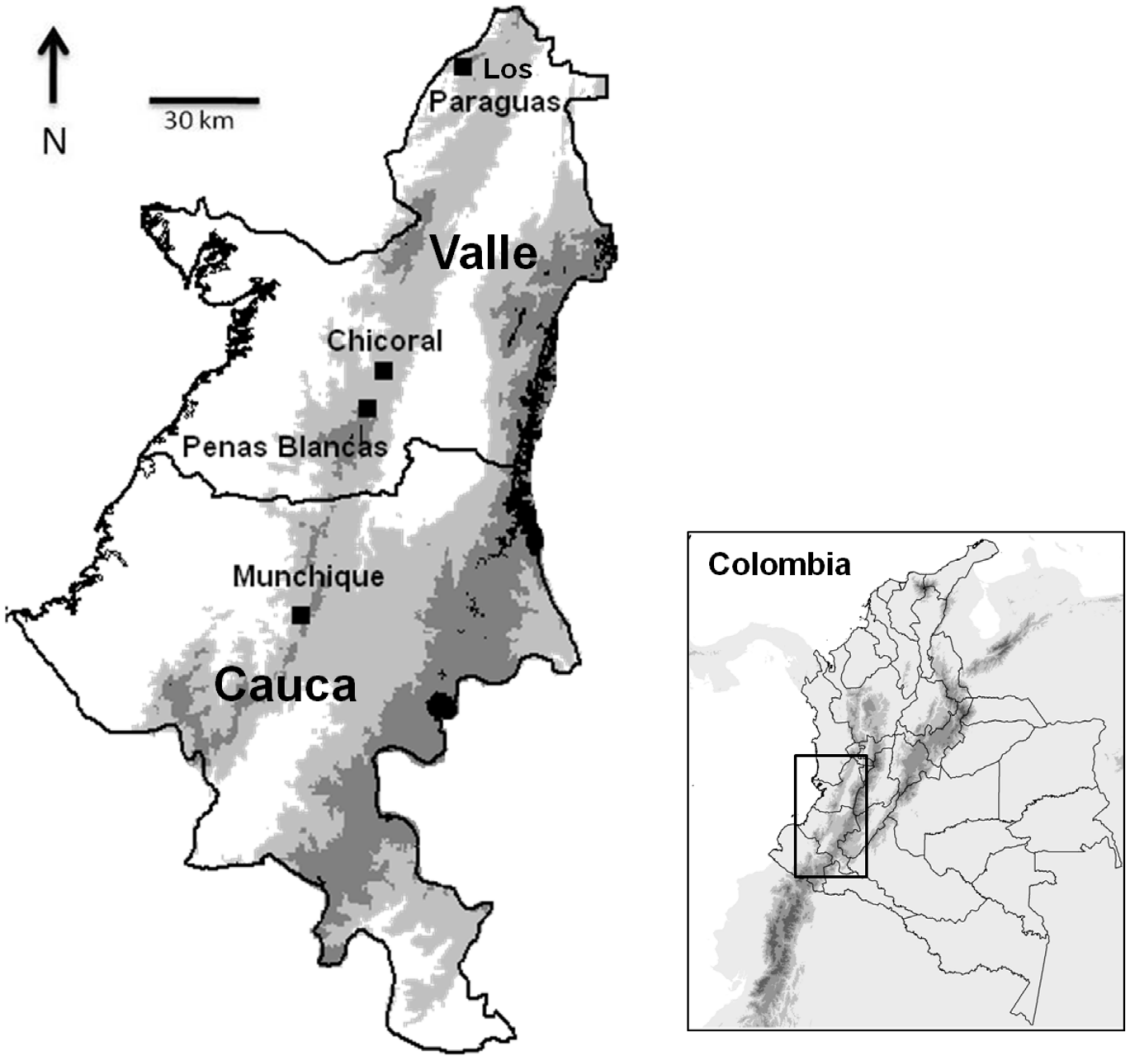
Map of western Colombia showing the four sampling localities in the departments of Cauca and Valle del Cauca.

Haplotype relationships were illustrated in a median-joining network (Fig. 1). The two sampling localities for *P. brevifrons* (Los Paraguas and Peñas Blancas), located 151.8 km apart and about at roughly same altitude, did not share haplotypes (each locality contained unique haplotypes) either in 16S or COI, which is consistent with a long period of isolation. As expected from the geography (Fig. 2), for *P. palmeri* the Serranía de Los Paraguas had the greatest differentiation as compared to the other two more proximal localities (Peñas Blancas and Chicoral) (Fig. 1). Chicoral and Peñas Blancas sites are separated by 18 km, while the northern population of Los Paraguas is more than 134 km apart from those sites.

There was a highly divergent COI haplotype found in two specimens (UVC15845 and UVC15857) within Peñas Blancas population of *P. palmeri* that were 17% (p-distance) diverged from what were presumably conspecific samples found at the same site. We were unable to amplify 16S from these same two specimens, unfortunately. Furthermore, there was one haplotype from the Chicoral population closely related to samples found at Peñas Blancas, indicating recent gene flow between these populations (Fig. 1). *Pristimantis jubatus* showed relatively low mtDNA divergences among sampling sites, with a total of four haplotypes for both genes. One individual of *P. jubatus* (UVC15842) from the site Observatorio was highly diverged (4% and 8%, p-distances for 16S and COI, respectively) from its conspecifics, with 22 nucleotide differences at 16S and 46 differences at COI from others at the same locality (Fig. 1). These divergence haplotypes within *P. palmeri* and *P. jubatus* may be migrants from an unsampled locality or cryptic species considering the intra-specific threshold values of divergence proposed by Vences et al. [37] and Fouquet et al. [38] of 3–5% at 16S and 10% at COI.

### Population Genetic Structure

For two of the three species, mtDNA variation showed patterns of population subdivision. Population genetic structure was high among localities for the widespread, generalist species *P. palmeri* and *P. brevifrons* (Table 1). For *P. palmeri* the minimum estimated pairwise *F*_ST_ among populations was found between Chicoral and Peñas Blancas (*F*_ST_ = 0.682, probability of the null hypothesis of panmixa, *p*<0.01), which are geographically closer. The greatest population structuring was observed between Paraguas and Peñas Blancas (the two most distant sampling sites) in both *P. brevifrons* (*F*_ST_ = 0.98, *p*<0.01) and *P. palmeri* (*F*_ST_ = 0.95, *p*<0.01). However, substantial polymorphism within populations resulted in low but significant proportions of the total variation partitioned among localities in the AMOVA (29.9%, *p*<0.01 and 21.6%, *p*<0.01, for *P. brevifrons* and *P. palmeri*, respectively). In the more range-restricted and habitat-specialized species *P. jubatus*, in contrast, we observed low mtDNA sequences divergence among all three localities (*F*_ST_ = 0.0076–0.2749) and a low percentage of the total variation partitioned among localities (1.34%, *p*>0.05). These results suggest that the three sampling localities 3 km apart, Observatorio, Santa Ana and Charguayaco (Table 2) within the Munchique National Park (Fig. 2) form a single genetic population.

**Table 1:** *F*_ST_ estimated and confidence limits (in parentheses) between pairs of populations of *P. brevifrons* and *P. palmeri* using mtDNA sequences from the 16S and COI genes combined.

Species	Locality	Chicoral	Peñas Blancas
*P. brevifrons*	Paraguas		0.9840 (0.976–1.000)
*P. palmeri*	Paraguas	0.9387 (0.896–0.982)	0.9506 (0.910–0.982)
	Chicoral		0.6821 (0.468–0.894)

**Table 2:** Sampling locations.

Locality	Taxon	N	Altitude (masl)	Transect (km)*	Description of site
Los Paraguas	*P. brevifrons*	7	1950–2050	0.8	Primary forest
Peñas Blancas	*P. brevifrons*	12	2000–2200	1.4	Secondary forest
Los Paraguas	*P. palmeri*	7	1950–2200	1.0	Primary forest
Chicoral	*P. palmeri*	15	1800–1850	0.8	Secondary forest
Peñas Blancas	*P. palmeri*	13	1950–2050	1.2	Secondary forest
Observatorio	*P. jubatus*	13	2550	0.2	Primary forest
Santa Ana	*P. jubatus*	8	2700	0.3	Primary forest
Charguayaco	*P. jubatus*	10	2450	0.2	Primary forest

* Transect refers to the straight distance along which samples were collected at each site. The three sites for *P. jubatus* are contained within the Munchique National Park (Fig. 2).

Tajima’s D statistic for *P. jubatus* for the combined Observatorio-Santa Ana-Charguayaco data (treated as one population, based on the above results), showed a significantly site-frequency skew for the mitochondrial dataset (D_T_ ≤ - 2.5 for both genes, Table 3). These values were due to the sample UVC15842 (found in Observatorio, see above), which increased the number of singleton sites and caused a rejection of the standard neutral model. If this sample was excluded, neutrality was not rejected (Table 3). Summary statistics of genetic polymorphisms for all populations of the three species of *Pristimantis* are provided in Table 3.

**Table 3:** Genetic polymorphism data for mitochondrial DNA sequences from the *Pristimantis* populations in this study.

Loci	Species	Population	N	h	S	*θ_W_*	π	D_T_	D_T_ 95% I.C.	Prob. D_T_≠0 Simul. Coal.
16S	*P. brevifrons*	Paraguas	7	2	1	0.00076	0.00053	−1.0062	−1.0062 to 1.341	0.490
		Peñas Blancas	12	4	3	0.00186	0.00094	−1.6292	−1.629 to 1.972	0.543
	*P. jubatus*	Observatorio-Santa Ana-Charguayaco	30	4	19	0.01047	0.0029	−2.5212*/−**1.248**	−1.712 to 1.816/−**1.509 to 2.014**	0.000
	*P. palmeri*	Paraguas	7	1	0	0	0	N.A.	N.A.	N.A.
		Chicoral	14	4	4	0.00235	0.00152	−1.1644	−1.797 to 1.886	0.1440
		Peñas Blancas	11	1	0	0	0	N.A.	N.A.	N.A.
COI	*P. brevifrons*	Paraguas	6	2	3	0.00201	0.00153	−1.2331	−1.233 to 1.909	0.268
		Peñas Blancas	10	2	1	0.00057	0.00032	−1.1117	−1.111 to 1.463	0.412
	*P. jubatus*	Observatorio-Santa Ana-Charguayaco	31	4	48	0.02036	0.00604	−2.6023*/**0.2368**	−1.719 to 1.792/−**1.507 to 2.027**	0.000
	*P. palmeri*	Paraguas	7	3	2	0.00132	0.0017	1.1684	−1.237 to 1.649	0.522
		Chicoral	15	7	10	0.00498	0.0045	−0.3644	−1.802 to 1.827	0.554
		Peñas Blancas	13	5	94	0.04901	0.04527	−0.3921	−1.760 to 1.700	0.543

N  =  number of sequences, h  =  number of haplotypes, S  =  segregating sites, *θ_W_*  =  population mutation rate per site, π  =  nucleotide diversity per site, D_T_  =  Tajima’s D statistic [73]. An asterisk (*) in the D_T_ column indicates a significant deviation from the expected value under neutrality (D_T_ = 0) as calculated from coalescence simulations. D_T_ values in **bold** obtained for *P. jubatus* when the divergent sample UVC15842 was excluded. N.A.  =  Not Applicable. Indels and sites with missing data were not taken into account in pairwise comparisons.

### Estimation of Historical Demographic Parameters

Gene flow (*Nm*) since initial divergence was very low between the two sampling localities of *P. brevifrons*, and within *P. palmeri* between the northerly Los Paraguas and the two southern sites. In contrast, significant and asymmetric gene flow was detected between the two adjacent populations of *P. palmeri*, with migration from Chicoral into Peñas Blancas being lower than in the reverse direction (Table 4, Fig. S1).

**Table 4:** Estimates obtained from IMa for the population size parameter theta (*N_e_*), gene flow (*Nm*) and divergence times (*t*) and 90% highest posterior density (HPD) intervals for populations of *P. brevifrons* (one pairwise comparison among two localities) and *P. palmeri* (three pairwise comparisons among three localities), based on the combined COI and 16S data.

Species	Samples	Parameter				
		*Ne*_1_	*Ne*_2_	*N*_1_ *m*_1_	*N*_2_ *m*_2_	*t*, years
*P. brevifrons*	Paraguas/Peñas Blancas	38595	68147	0.0006	0.0009	802500
	90% HPD	6056–149013	21238–181175	0.0001–1.6	0.0002–1.6	565227–1362954*
*P. palmeri*	Paraguas/Peñas Blancas	8697	52638	0.0002	0.0009	584545
	90% HPD	486–59206	16184–145029	0.00001–1.1	0.0002–1.8	406363–1817272*
	Paraguas/Chicoral	12572	143393	0.0004	0.001	541818
	90% HPD	813–73170	69452–280711	0.00003–1.5	0.0009–2.3	344000–1453818*
	Chicoral/Peñas Blancas	102922	46075	1.2	0.004	146818
	90% HPD	39411–240720	11765–133970	0.003–13.9	0.001–10.0	67727–908636*

* indicates that HPD estimates are unreliable because the tail of the distribution did not reach zero.

Based on estimates of *θ* for the combined mitochondrial gene sequences and using an inheritance scalar of 0.25 and a mutation rate estimated from closely related frogs (see Methods), we calculated an effective population size (*N_e_*) for *P. brevifrons* of 38,000 (90% HPD 6,000–150,000) and 68,000 (90% HPD 21,000–181,000) reproductive individuals in Paraguas and Peñas Blancas, respectively. For *P. palmeri* we estimated *N_e_* at 10,000 (90% HPD 500–73,000) individuals in Paraguas, 50,000 (90% HPD 12,000–145,000) for Peñas Blancas and 100,000 (90% HPD 40,000–280,000) at Chicoral (Table 4, Fig. S2). To be more conservative in our estimates of *N*_e_ we removed the unusually divergent specimens of *P. palmeri* (UVC15857 and UVC15845) from Peñas Blancas. Including these samples would inflate the number of segregating sites and bias estimates of *N*_e_ upwards. Because the three collecting localities for *P. jubatus* apparently represented a single genetic population, their effective population size was obtained using MIGRATE [39]. As the specimen UVC15842 of *P. jubatus* caused significant departures from standard neutral expectations (see above), we removed this sample prior to calculating *N*_e_, yielding a more conservative estimated effective population size for this species of 94,000 (95% C.I. 15,600–470,000) individuals.

The upper tail of the posterior distribution for divergence times did not reach zero probability so we were unable to estimate a strict 90% HPD value for this parameter for any species. The partial posterior distributions, however, suggested the following tentative approximations of divergence times. The mode of the posterior distribution of divergence time among *P. palmeri* populations was about 0.58 million years ago (Mya) (90% HPD roughly 0.40–1.8 Mya) between Paraguas and Peñas Blancas, and divergence time between adjacent populations Chicoral and Peñas Blancas was 0.14 Mya (0.06–0.90 Mya) (Table 4, Fig. S3). Divergence times across the same terrain (Paraguas to Peñas Blancas) were slightly older in *P. brevifrons* (0.80 Mya; 90% HPD 0.56–1.4 Mya) than in *P. palmeri* (0.58).

## Discussion

Studies on the intraspecific genetic differentiation and population structure of anurans in montane regions are sorely needed, considering that work in montane areas lags behind work done in low elevation environments of the Neotropics (e.g. Central America and Amazonia) [40], [41], [42]. We argue here that much interspecific diversity was likely derived from within-species diversity through vicariance processes, and these processes in term were actively promoted by millions of years of remarkable geological dynamism of the Andean Cordillera [21], [40]. Thus, an understanding of interspecific diversity patterns is informed by the study of within-species historical demographic processes [40], [43].

This study characterizes the genetic diversity, population structure and phylogeography of *P. brevifrons*, *P. palmeri* and *P. jubatus* using the mitochondrial markers COI and 16S. Estimates of gene flow, population sizes, and divergence time revealed important aspects of the historical demography of these three Neotropical montane species. Our data highlighted the undifferentiated structure of *P. jubatus* at a local geographical scale as well as the similar phylogeographic structure in *P. brevifrons* and *P. palmeri*, two species with similar life histories.

### Comparative Phylogeographic of *Pristimantis* Frogs

For *P. brevifrons* and *P. palmeri* analyses of the two mitochondrial loci revealed high genetic differentiation in space cross the same geographic scale landscape (approx. 150 km.) and a similar inferred age of population differentiation in both species (around 0.80 Mya) (Fig. 1, Table 4). This implies that the two widespread species responded similarly to shared environmental processes producing similar population histories at the same spatial scale. Because these species are similar in terms of their ecological requirements, their parallel phylogeographic patterns suggest an eco-geological explanation of the geographical pattern during the Pleistocene [18], [19], [44].

The northern population of Los Paraguas showed low migration rates with the two southern sites, and a corresponding high pairwise population differentiation. This population appears to have split from the other locations during the Mid-Pleistocene (0.58–0.80 Mya), i.e., following the last episode of uplift of the northern portion of the Andes in the Pliocene (5.0–2.0 Mya) [45], [46]. The Serranía de Los Paraguas is a mountain formation that follows the western flank of the Cordillera Occidental, diverging from the main mountain range. This geological separation may be the cause of the restricted gene flow with the southern localities, thereby promoting the divergence of the Los Paraguas population. In the Cordillera Oriental of Colombia a similar pattern of isolation by ridges is found in unrelated frog species [9], [47].

Despite the dearth of ecological data for species of *Pristimantis*, these frogs are thought to have small home ranges along with low vagility [29], [48], which is supported by genetic data in a few species [40], [42]. At a smaller spatial scale (∼3 km), our analysis for the narrowly distributed *P. jubatus,* showed no significant spatial differentiation. We expected that the habitat specialist *P. jubatus* would show high structure but we likely need more geographically extensive sampling. On the other hand, we might have expected direct-developing frogs to show minimal population structuring as these amphibians do not require a water source for ovipositing or mating, so they may breed anywhere within a continuous forest of high relative humidity [40]. *Pristimantis palmeri* perhaps fits this prediction an intermediate spatial scale, i.e., the 18 km between Chicoral and Peñas Blancas. We estimated the asymmetric migration rates at 1.2 (90% HPD 0.003–13.9) and 0.004 (0.001–10.0), thus we could not reject the hypothesis of substantial levels of gene flow at this distance.

To date, there is a limited yet growing number of studies on genetic structure at local or regional scales in Neotropical frogs e.g., [40], [42], [49]. In other studies of terraranid frogs, Elmer et al. [42] found genetic structure at 4 km but Crawford [40] found no mitochondrial or nuclear differentiation between populations separated by 10 km. Also, Guarnizo et al. [9] found identical DNA sequences in localities as far away as 26 km in the high-elevation Andean hylid frog, *Dendropsophus labialis*. These results indicate that Andean frogs may disperse long distances at least in some cases or in some geographic context [27] and their range limits may be perhaps closely associated to altitude due to thermal limitations [50], [51], that may increase opportunities for speciation and high beta diversity in the Andes [52].

Our IMa analyses produced robust historical demographic parameter estimates, with the exception of divergences times. Features of the evolutionary history of our three study species revealed by coalescent analyses are particularly informative because few data on historical demography are currently available for tropical amphibians. Demographic estimates of population size are not available for *P. brevifrons, P. palmeri* or *P. jubatus* to corroborate the genetic-based estimates, however, the estimated population sizes for *P. brevifrons, P. palmeri* and *P. jubatus* are quite similar compared to those obtained by Crawford [40] for the terraranid species *Craugastor bransfordii* (3.1×10^5^) and *C. stejnegerianus* (1.0×10^5^). Thus, the effective population sizes are potentially very large in our three species of *Pristimantis*. Large population sizes could be positively correlated to higher speciation rates [53], yet a more parsimonious explanation may be that the beta diversity of *Pristimantis* in montane regions has been promoted by barriers, such as the Peñas Blancas ridge. Our results here inform these biogeographic models by confirming that even localized and isolated populations of these relatively small frogs may hold substantial amounts of genetic diversity, thus contributing to the maintenance of viable populations through subsequent vicariant events during montane diversification [23]. MtDNA lineages within *Pristimantis* frogs showed signs of demographic changes associated with recent Pleistocene and geological events. It will be interesting to examine similarly widespread species to evaluate the generality of these patterns and conduct more fine-scaled phylogeographical analyses to explore the possible mechanisms leading to speciation within this group of direct-developing frogs.

## Materials and Methods

### Ethics Statement

This study was carried out in strict accordance with the evaluations of environmental, social, and educational impacts established for research projects under Universidad del Valle *Convocatoria Interna* number CI-746. Permit for sampling of specimens was obtained from Ministerio de Medio Ambiente, Vivienda y Desarrollo Territorial de Colombia (Resolución 573 del 10 de abril/08). Euthanasia of frogs was performed with Chloretone, and all efforts were made to minimize suffering. Specimens were deposited at Herpetological Collection – Universidad del Valle (Voucher numbers: UVC15812– UVC15954) (Table S1) and DNA sequences data have been submitted to Genbank: accession numbers JN104663– JN104683; JN370956– JN371118.

### Sampling

Sampling was carried out at four localities (Fig. 2): La Serranía de Los Paraguas, municipality El Cairo, on the border of the departments of Valle del Cauca and Chocó, 2,000–2,200 masl (4.7333°, −76.3°); Chicoral, municipality La Cumbre, department of Valle del Cauca, 1,800–1,900 masl (3.5833°, −76.6°); Peñas Blancas, in Farallones de Cali National Park, municipality Cali, department of Valle del Cauca, 1,900–2,200 masl (3.4333°, −76.6667°); and Munchique National Park, municipality El Tambo, department of Cauca, 2,400–2,800 masl (2.6667°, −76.9167°).

At each locality we established approximately straight-line transects across which samples were collected. Samples sizes ranged from 7–15 individuals per location (Table 2). *Pristimantis jubatus* was sampled at three different locations in the Munchique National Park (Observatorio, Santa Ana and Charguayaco), while widespread, generalist species *P. brevifrons* and *P. palmeri* were sampled at La Serranía de Los Paraguas and Peñas Blancas, and Serranía de Los Paraguas, Peñas Blancas and Chicoral, respectively. Tissues were collected in the field and kept in a solution of 1 M Tris HCl, 0.5 M EDTA, 5 M NaCl and SDS to 20%. Specimen identification was confirmed by Dr. John D. Lynch, Curator of Amphibians at the Instituto de Ciencias Naturales, Universidad Nacional de Colombia, Bogotá.

### Laboratory Techniques

Total DNA was extracted from liver or thigh tissue using the Qiagen Tissue DNeasy kit (Qiagen) following the manufacturer’s instructions. Mitochondrial DNA (mtDNA) fragments were amplified using PCR primers 16 Sbr plus 16 Sar for the ARN region of the 16S ribosomal RNA gene [54] and LCO1490 plus HCO2198 for the 5′ half of the cytochrome oxidase subunit I (COI), also known as the Folmer fragment or Barcode of Life fragment for animals [55], [56]. The DNA amplification reactions contained 2.5 mM MgCl_2_, 0.3 mM dNTPs, 0.3 µM of each Primer, 0.625 U of Taq polymerase and 1–2 µl total DNA. PCR conditions followed Goebel et al. [57] and Vences et al. [58]. Negative controls were used to monitor potential contamination in PCR. Amplification products were purified with QIAquick PCR clean-up kit (Qiagen). Both H (heavy) and L (light) strands were sequenced using the chemical reaction Big-Dye Terminator v. 3.1 reagents in an automated DNA sequencer (ABI 3730XL, Applied Biosystems Inc.). The sequences were deposited in GenBank (Table S1).

### Analyses

#### DNA sequence analysis

Sequences were edited, assembled and aligned using the program Sequencher 4.6 (Gene Codes Corporation) and verified by eye. COI sequences were translated into amino acids with the program DnaSP 5.0 [59] to confirm the absence of inferred nonsense mutations. We used DnaSP to calculate the following summary statistics for genetic variation within each population: haplotype diversity (Hd), nucleotide diversity per site (*π* ) [60], the number of segregating sites (*S*), Watterson’s estimator of the per-site population mutation rate (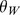) [61] and Tajima’s D statistic (*D*_T_). Sites with indels or missing data were eliminated in pairwise sequence comparisons. Coalescence simulations were run in DnaSP to test for significant departures from the expected value *D*_T_ = 0 under the standard neutral model using 5,000 replicates conditional on sample size and *S*.

#### Analysis of population differentiation

We used the programs Arlequin 3.1 [62] and DnaSP 5.0 to conduct an Analysis of Molecular Variance (AMOVA) and calculate Lynch & Crease’s pairwise *F*_ST_ [63] to test the null hypothesis of panmixia between pairs of populations [64] using 1000 permutations and significance level of 0.05. *F*_ST_ was chosen as the metric parameter of the genetic differentiation among populations because it provides a more effective summary of the effects of population structure [65]. To visualize the relationship between the genealogy of haplotypes and geography, haplotype networks were inferred using median joining as implemented in the program Network 4.5.1.0 [66].

#### Estimation historical demographic parameters

We estimated the effective population sizes (*N*_e_), asymmetric pairwise migration rates (*Nm*) and time since population divergence (*t*) with the programs IMa [67] and MIGRATE [39]. To convert parameter estimates of IMa into demographic units, an inheritance scalar of 0.25 and generation time of one year were assumed. To obtain *N*_e_ from *θ* ( = 4*N*_e_ *μ*, under the standard neutral model) we assumed a mutation rate, *μ*, of 1.9×10^−8 ^per site per year, based on estimated rates of silent site divergence in a genus of frogs closely related to *Pristimantis* [68]. While the COI gene typically has higher overall rates of substitution relative to the more conserved 16S gene [37], [69], [70], we assume that these two mitochondrial genes experience comparable rates of mutation [71] (as opposed to substitution rates). We used an infinite-site model of substitution [72] for both genes, 16S and COI. Several preliminary MCMC simulations with broad priors were performed to set appropriate bounds for each parameter (*θ, m, t*). We compared the convergence of the marginal distributions of each parameter among multiple runs and obtained the credibility intervals based on 90% highest posterior density (HPD).

## Supporting Information

Figure S1 Posterior probability distributions for gene flow from IMa analysis.
(TIF)

Figure S2 Posterior probability distributions for effective population sizes from IMa analysis.
(TIF)

Figure S3 Posterior probability distributions for divergence times from IMa analysis.
(TIF)

Table S1 Sample information for taxa analyzed in this study.
(DOC)
