# Supplementary figures and images for: Comparative Phylogeography of Direct-Developing Frogs (Anura: Craugastoridae: Pristimantis) in the Southern Andes of Colombia
Source: PLoS One. 2012 Sep 25;7(9):e46077. doi: 10.1371/journal.pone.0046077 (PMC3457947; doi:10.1371/journal.pone.0046077)

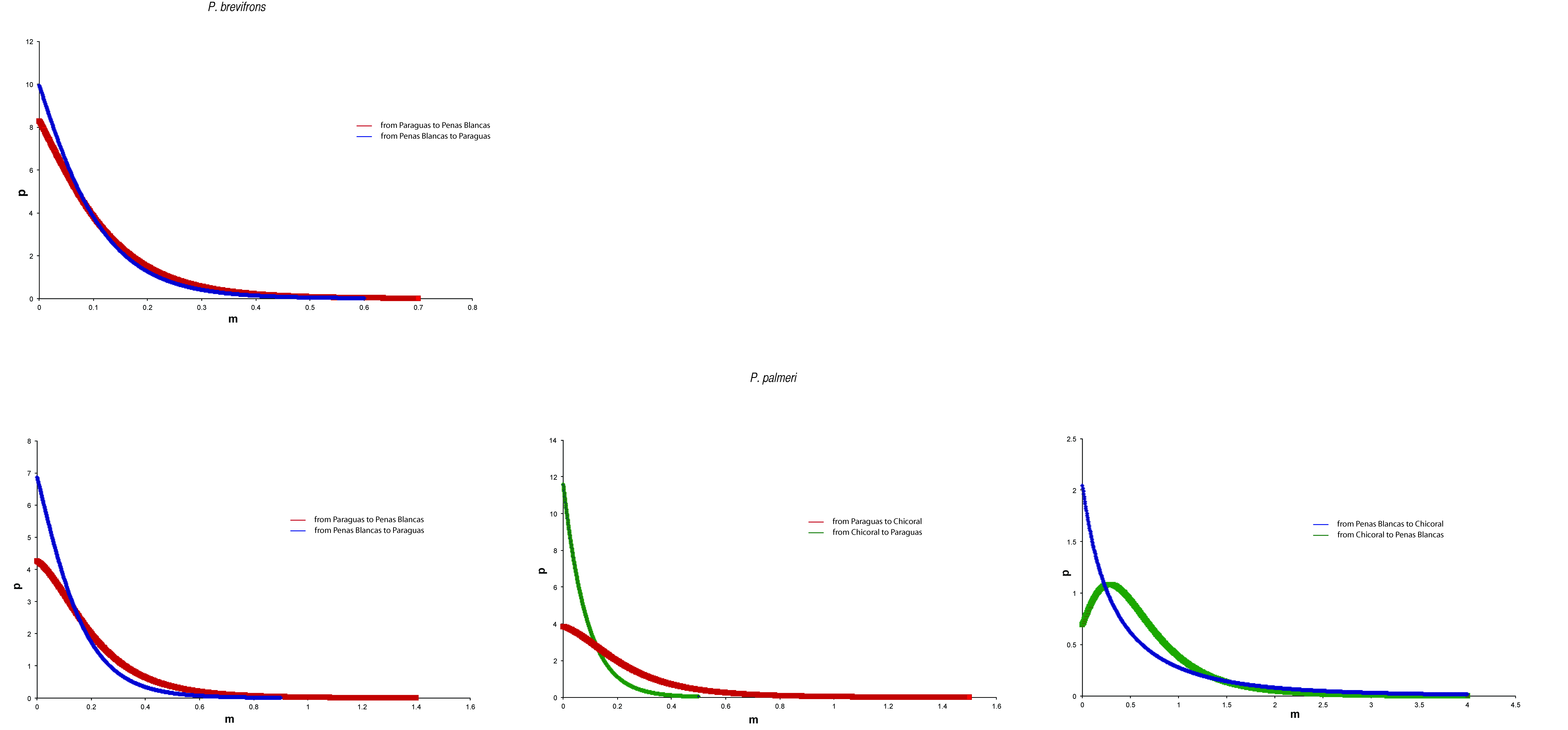

Supplement: Figure S1 — Posterior probability distributions for gene flow from IMa analysis. (TIF) [file pone.0046077.s001.tif]

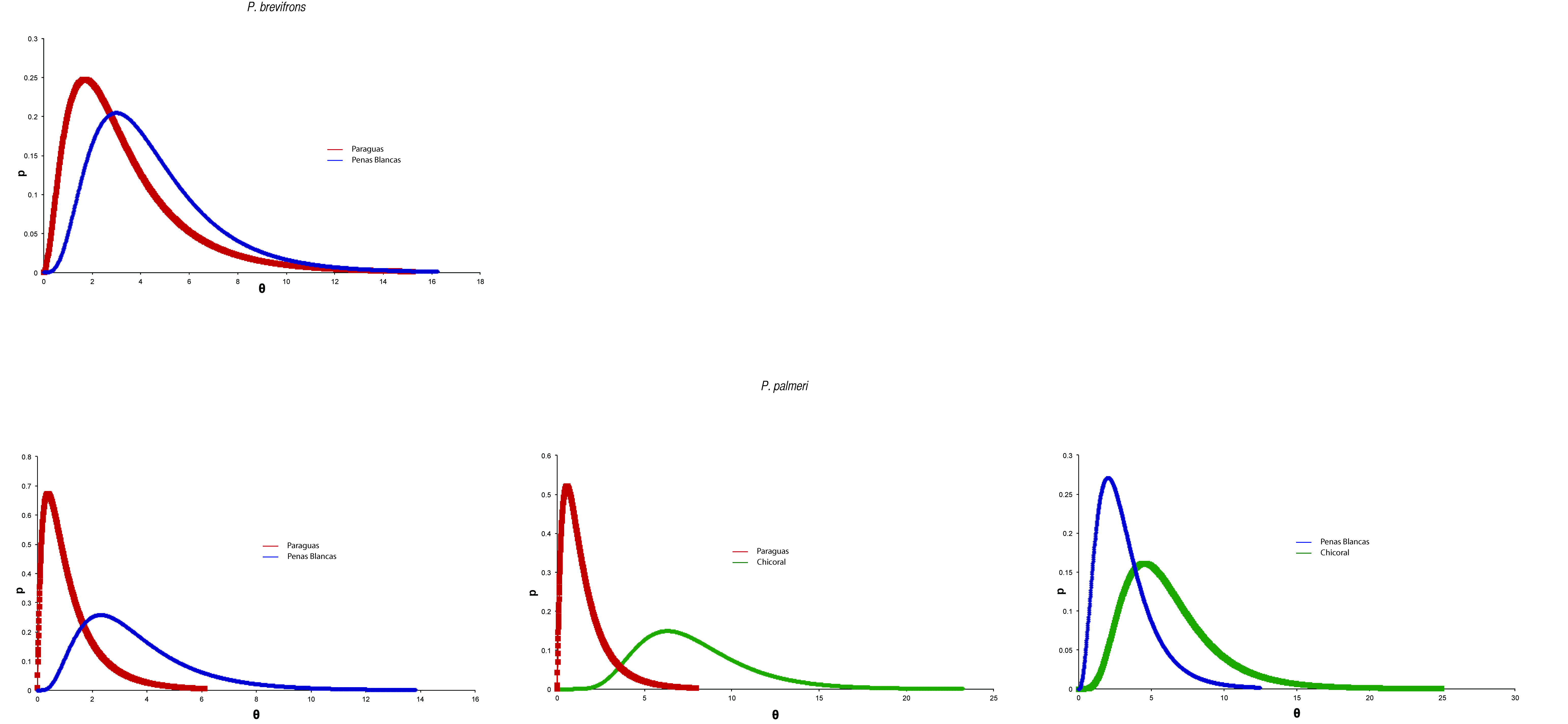

Supplement: Figure S2 — Posterior probability distributions for effective population sizes from IMa analysis. (TIF) [file pone.0046077.s002.tif]

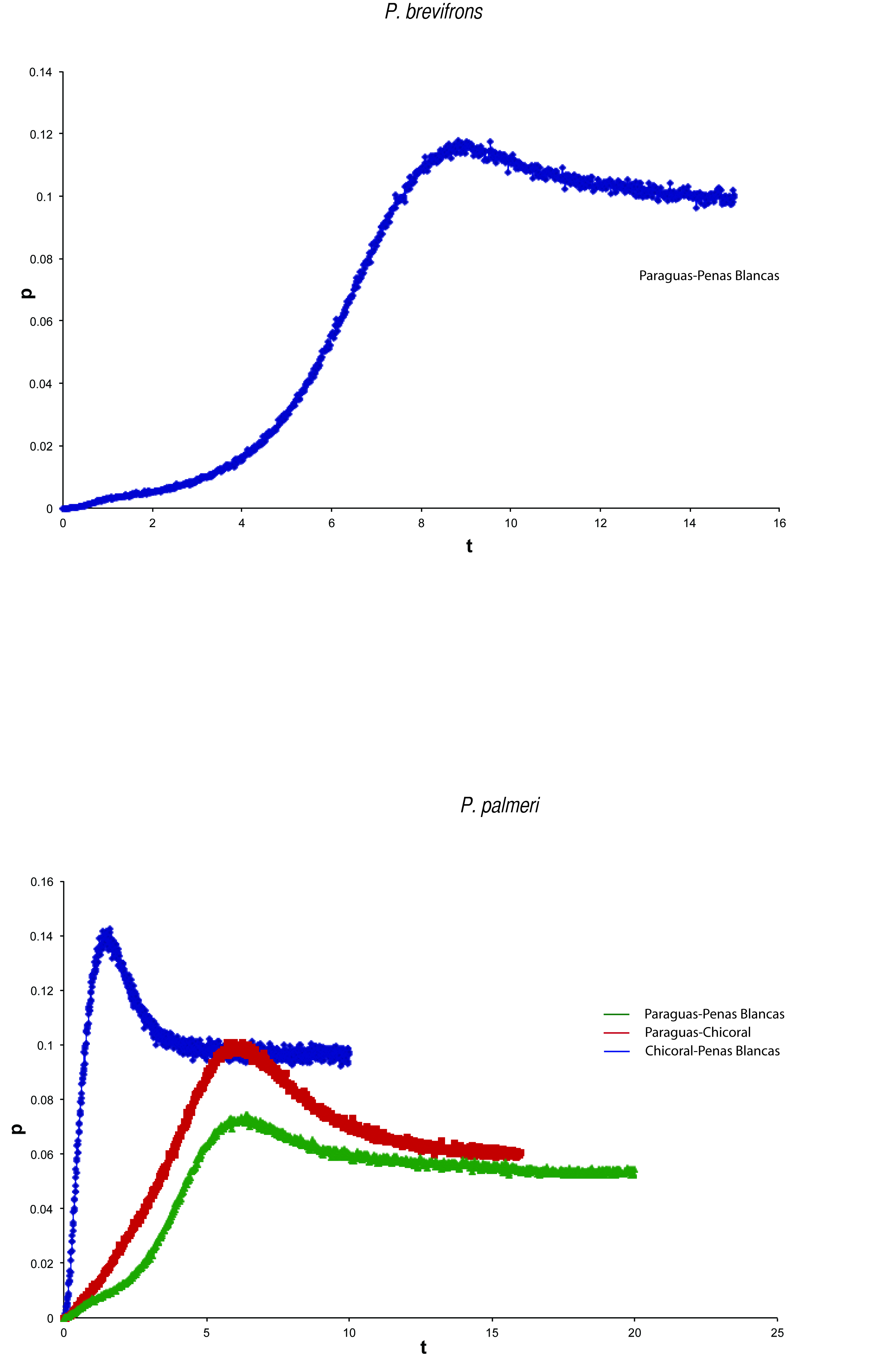

Supplement: Figure S3 — Posterior probability distributions for divergence times from IMa analysis. (TIF) [file pone.0046077.s003.tif]
