# Supplementary material for: Comparative Phylogeography of Direct-Developing Frogs (Anura: Craugastoridae: Pristimantis) in the Southern Andes of Colombia
Source: PLoS One. 2012 Sep 25;7(9):e46077. doi: 10.1371/journal.pone.0046077 (PMC3457947; doi:10.1371/journal.pone.0046077)
Supplement: Table S1 — Sample information for taxa analyzed in this study. (DOC) [file pone.0046077.s004.doc]

**Supporting Information**

Table S1. Sample information for taxa analyzed in this study.

| Taxon and museum voucher | Locality | Geographical coordinates | | Altitude | GenBank Accession Numbers | |
| --- | --- | --- | --- | --- | --- | --- |
|  |  | N | W |  | 16S | COI |
| *P. brevifrons* | | | | | | |
| UVC 15858 | Serrania de Los Paraguas | 4º 44' 33.9'' | 76º 17' 45.0'' | 2050 m | JN370956 | JN371040 |
| UVC 15856 | Serrania de Los Paraguas | 4º 44' 33.9'' | 76º 17' 45.0'' | 2050 m | JN370957 | JN371041 |
| UVC 15896 | Serrania de Los Paraguas | 4º 44' 33.9'' | 76º 17' 45.0'' | 2050 m | JN370958 |  |
| UVC 15898 | Serrania de Los Paraguas | 4º 44' 33.9'' | 76º 17' 45.0'' | 2050 m | JN370959 | JN371042 |
| UVC 15826 | Serrania de Los Paraguas | 4º 44' 33.9'' | 76º 17' 45.0'' | 2050 m | JN370960 | JN371043 |
| UVC 15852 | Serrania de Los Paraguas | 4º 44' 33.9'' | 76º 17' 45.0'' | 2050 m | JN370961 | JN371044 |
| UVC 15834 | Serrania de Los Paraguas | 4º 44' 33.9'' | 76º 17' 45.0'' | 2050 m | JN370962 | JN371045 |
| UVC 15909 | Farallones de Cali | 3º 25' 43.9'' | 76º 39' 33.4'' | 2100 m | JN370963 | JN371046 |
| UVC 15831 | Farallones de Cali | 3º 25' 43.9'' | 76º 39' 33.4'' | 2100 m | JN370964 | JN371047 |
| UVC 15910 | Farallones de Cali | 3º 25' 43.9'' | 76º 39' 33.4'' | 2100 m | JN370965 | JN371048 |
| UVC 15829 | Farallones de Cali | 3º 25' 43.9'' | 76º 39' 33.4'' | 2100 m | JN370966 | JN371049 |
| UVC 15833 | Farallones de Cali | 3º 25' 43.9'' | 76º 39' 33.4'' | 2100 m | JN370967 | JN371050 |
| UVC 15825 | Farallones de Cali | 3º 25' 43.9'' | 76º 39' 33.4'' | 2100 m | JN104678 | JN371051 |
| UVC 15908 | Farallones de Cali | 3º 25' 43.9'' | 76º 39' 33.4'' | 2100 m | JN370968 | JN371052 |
| UVC 15841 | Farallones de Cali | 3º 25' 43.9'' | 76º 39' 33.4'' | 2100 m | JN370969 | JN371053 |
| UVC 15912 | Farallones de Cali | 3º 25' 43.9'' | 76º 39' 33.4'' | 2100 m | JN370970 | JN371054 |
| UVC 15844 | Farallones de Cali | 3º 25' 43.9'' | 76º 39' 33.4'' | 2100 m | JN370971 |  |
| UVC 15885 | Farallones de Cali | 3º 25' 43.9'' | 76º 39' 33.4'' | 2100 m | JN370972 |  |
| UVC 15904 | Farallones de Cali | 3º 25' 43.9'' | 76º 39' 33.4'' | 2100 m | JN370973 | JN371055 |
| *P. palmeri* | | | | | | |
| UVC 15913 | Serrania de Los Paraguas | 4º 47' 11.8'' | 76º 13' 54.7'' | 2160 m | JN371001 | JN371084 |
| UVC 15836 | Serrania de Los Paraguas | 4º 47' 11.8'' | 76º 13' 54.7'' | 2160 m | JN371002 | JN371085 |
| UVC 15823 | Serrania de Los Paraguas | 4º 44' 33.9'' | 76º 17' 45.0'' | 2050 m | JN371003 | JN371086 |
| UVC 15814 | Serrania de Los Paraguas | 4º 44' 33.9'' | 76º 17' 45.0'' | 2050 m | JN104683 | JN371087 |
| UVC 15862 | Serrania de Los Paraguas | 4º 44' 33.9'' | 76º 17' 45.0'' | 2050 m | JN371004 | JN371088 |
| UVC 15821 | Serrania de Los Paraguas | 4º 44' 33.9'' | 76º 17' 45.0'' | 2050 m | JN371005 | JN371089 |
| UVC 15832 | Serrania de Los Paraguas | 4º 48' 08.0'' | 76º 09' 00.0'' | 1750 m | JN371006 | JN371090 |
| UVC 15921 | Bitaco | 3º 34' 28.4'' | 76º 35' 48.5'' | 1850 m | JN371007 | JN371091 |
| UVC 15830 | Bitaco | 3º 34' 28.4'' | 76º 35' 48.5'' | 1850 m | JN371008 | JN371092 |
| UVC 15822 | Bitaco | 3º 34' 28.4'' | 76º 35' 48.5'' | 1850 m | JN371009 | JN371093 |
| UVC 15891 | Bitaco | 3º 34' 28.4'' | 76º 35' 48.5'' | 1850 m | JN371010 | JN371094 |
| UVC 15839 | Bitaco | 3º 34' 28.4'' | 76º 35' 48.5'' | 1850 m | JN371011 | JN371095 |
| UVC 15820 | Bitaco | 3º 34' 28.4'' | 76º 35' 48.5'' | 1850 m | JN371012 | JN371096 |
| UVC 15907 | Bitaco | 3º 34' 28.4'' | 76º 35' 48.5'' | 1850 m | JN371013 | JN371097 |
| UVC 15859 | Bitaco | 3º 34' 28.4'' | 76º 35' 48.5'' | 1850 m |  | JN371098 |
| UVC 15840 | Bitaco | 3º 34' 28.4'' | 76º 35' 48.5'' | 1850 m | JN371014 | JN371099 |
| UVC 15882 | Bitaco | 3º 34' 28.4'' | 76º 35' 48.5'' | 1850 m | JN371015 | JN371100 |
| UVC 15887 | Bitaco | 3º 34' 28.4'' | 76º 35' 48.5'' | 1850 m | JN371016 | JN371101 |
| UVC 15869 | Bitaco | 3º 34' 28.4'' | 76º 35' 48.5'' | 1850 m | JN371017 | JN371102 |
| UVC 15835 | Bitaco | 3º 34' 28.4'' | 76º 35' 48.5'' | 1850 m | JN371018 | JN371103 |
| UVC 15900 | Bitaco | 3º 34' 28.4'' | 76º 35' 48.5'' | 1850 m | JN371019 | JN371104 |
| UVC 15922 | Bitaco | 3º 34' 28.4'' | 76º 35' 48.5'' | 1850 m | JN371020 | JN371105 |
| UVC 15827 | Farallones de Cali | 3º 25' 43.9'' | 76º 39' 33.4'' | 2100 m | JN371021 | JN371106 |
| UVC 15851 | Farallones de Cali | 3º 25' 43.9'' | 76º 39' 33.4'' | 2100 m | JN371022 | JN371109 |
| UVC 15849 | Farallones de Cali | 3º 25' 43.9'' | 76º 39' 33.4'' | 2100 m | JN371023 | JN371110 |
| UVC 15876 | Farallones de Cali | 3º 25' 43.9'' | 76º 39' 33.4'' | 2100 m | JN371024 | JN371111 |
| UVC 15855 | Farallones de Cali | 3º 25' 43.9'' | 76º 39' 33.4'' | 2100 m | JN371025 | JN371112 |
| UVC 15924 | Farallones de Cali | 3º 25' 43.9'' | 76º 39' 33.4'' | 2100 m | JN371026 | JN371113 |
| UVC 15850 | Farallones de Cali | 3º 25' 43.9'' | 76º 39' 33.4'' | 2100 m | JN371027 | JN371114 |
| UVC 15868 | Farallones de Cali | 3º 25' 43.9'' | 76º 39' 33.4'' | 2100 m | JN371028 | JN371115 |
| UVC 15853 | Farallones de Cali | 3º 25' 43.9'' | 76º 39' 33.4'' | 2100 m | JN371029 | JN371116 |
| UVC 15837 | Farallones de Cali | 3º 25' 43.9'' | 76º 39' 33.4'' | 2100 m | JN371030 | JN371117 |
| UVC 15906 | Farallones de Cali | 3º 25' 43.9'' | 76º 39' 33.4'' | 2100 m | JN371031 | JN371118 |
| UVC 15845 | Farallones de Cali | 3º 25' 43.9'' | 76º 39' 33.4'' | 2100 m |  | JN371107 |
| UVC 15857 | Farallones de Cali | 3º 25' 43.9'' | 76º 39' 33.4'' | 2100 m |  | JN371108 |
| *P. jubatus* | | | | | | |
| UVC 15815 | Munchique National Park | 2º 38' 17.8'' | 76º 54' 54.7'' | 2542 m | JN370975 | JN371058 |
| UVC 15866 | Munchique National Park | 2º 38' 17.8'' | 76º 54' 54.7'' | 2542 m | JN370976 | JN371059 |
| UVC 15865 | Munchique National Park | 2º 38' 17.8'' | 76º 54' 54.7'' | 2542 m | JN370977 | JN371060 |
| UVC 15890 | Munchique National Park | 2º 38' 17.8'' | 76º 54' 54.7'' | 2542 m | JN370978 | JN371061 |
| UVC 15877 | Munchique National Park | 2º 38' 17.8'' | 76º 54' 54.7'' | 2542 m | JN104663 | JN104672 |
| UVC 15838 | Munchique National Park | 2º 38' 17.8'' | 76º 54' 54.7'' | 2542 m | JN370979 | JN371062 |
| UVC 15818 | Munchique National Park | 2º 38' 17.8'' | 76º 54' 54.7'' | 2542 m | JN370980 | JN371063 |
| UVC 15854 | Munchique National Park | 2º 38' 17.8'' | 76º 54' 54.7'' | 2542 m | JN370981 | JN371064 |
| UVC 15911 | Munchique National Park | 2º 38' 17.8'' | 76º 54' 54.7'' | 2542 m | JN370982 | JN371065 |
| UVC 15894 | Munchique National Park | 2º 38' 17.8'' | 76º 54' 54.7'' | 2542 m | JN370983 | JN371066 |
| UVC 15901 | Munchique National Park | 2º 38' 47.3'' | 76º 54' 31.4'' | 2642 m | JN370985 | JN371068 |
| UVC 15903 | Munchique National Park | 2º 38' 47.3'' | 76º 54' 31.4'' | 2642 m | JN370986 | JN371069 |
| UVC 15893 | Munchique National Park | 2º 38' 47.3'' | 76º 54' 31.4'' | 2642 m | JN370987 | JN371070 |
| UVC 15860 | Munchique National Park | 2º 38' 47.3'' | 76º 54' 31.4'' | 2642 m | JN370988 | JN371071 |
| UVC 15919 | Munchique National Park | 2º 38' 47.3'' | 76º 54' 31.4'' | 2642 m | JN370989 | JN371072 |
| UVC 15884 | Munchique National Park | 2º 38' 47.3'' | 76º 54' 31.4'' | 2642 m | JN104664 | JN104673 |
| UVC 15920 | Munchique National Park | 2º 38' 47.3'' | 76º 54' 31.4'' | 2642 m | JN370990 | JN371073 |
| UVC 15813 | Munchique National Park | 2º 38' 47.3'' | 76º 54' 31.4'' | 2642 m | JN370991 | JN371074 |
| UVC 15899 | Munchique National Park | 2º 39' 51.4'' | 76º 54' 09.0'' | 2460 m | JN370992 | JN371075 |
| UVC 15812 | Munchique National Park | 2º 39' 51.4'' | 76º 54' 09.0'' | 2460 m | JN370993 | JN371076 |
| UVC 15902 | Munchique National Park | 2º 39' 51.4'' | 76º 54' 09.0'' | 2460 m | JN370994 | JN371077 |
| UVC 15880 | Munchique National Park | 2º 39' 51.4'' | 76º 54' 09.0'' | 2460 m | JN370995 | JN371078 |
| UVC 15847 | Munchique National Park | 2º 39' 51.4'' | 76º 54' 09.0'' | 2460 m | JN104665 | JN104674 |
| UVC 15816 | Munchique National Park | 2º 39' 51.4'' | 76º 54' 09.0'' | 2460 m | JN370996 | JN371079 |
| UVC 15864 | Munchique National Park | 2º 39' 51.4'' | 76º 54' 09.0'' | 2460 m | JN370997 | JN371080 |
| UVC 15892 | Munchique National Park | 2º 39' 51.4'' | 76º 54' 09.0'' | 2460 m | JN370998 | JN371081 |
| UVC 15915 | Munchique National Park | 2º 39' 51.4'' | 76º 54' 09.0'' | 2460 m | JN370999 | JN371082 |
| UVC 15917 | Munchique National Park | 2º 39' 51.4'' | 76º 54' 09.0'' | 2460 m | JN371000 | JN371083 |
| UVC 15916 | Munchique National Park | 2º 38' 17.8'' | 76º 54' 54.7'' | 2542 m | JN370974 | JN371056 |
| UVC 15926 | Munchique National Park | 2º 38' 17.8'' | 76º 54' 54.7'' | 2542 m |  | JN371057 |
| UVC 15842 | Munchique National Park | 2º 38' 17.8'' | 76º 54' 54.7'' | 2542 m | JN370984 | JN371067 |
|  | | | | | | |
